# Effects of probiotics, prebiotics, synbiotics and postbiotics on pediatric asthma: a systematic review

**DOI:** 10.3389/fnut.2025.1586129

**Published:** 2025-04-25

**Authors:** Die Fan, Jilei Hu, Ning Lin

**Affiliations:** Clinical Nutrition, The General Hospital of Western Theater Command, Chengdu, China

**Keywords:** pediatric asthma, probiotics, prebiotics, synbiotics, postbiotics, systematic review

## Abstract

**Background:**

Pediatric asthma, a prevalent chronic disease with rising global incidence, imposing substantial healthcare and socioeconomic burdens. Emerging evidence highlights the gut-lung axis as a pivotal therapeutic target, with microbiota dysbiosis implicated in immune dysregulation and airway hyperresponsiveness. This systematic review evaluated the efficacy and safety of probiotics, prebiotics, synbiotics, and postbiotics in pediatric asthma management.

**Methods:**

A comprehensive search of PubMed, Cochrane library, Web of Science, and Embase was conducted up to 2nd January 2025. Inclusion criteria encompassed randomized controlled trials (RCTs) evaluating the therapeutic use of probiotics, prebiotics, synbiotics, or postbiotics in children and/or adolescents (<18 years) with asthma.

**Results:**

Eighteen studies (13 RCTs, *n* = 2,419 participants) were analyzed, focusing on children aged < 18 years. Probiotic interventions, predominantly *Lactobacillus* (5 studies) and *Bifidobacterium* (5 studies), demonstrated significant reductions in asthma exacerbations and improved pulmonary function, with strain-specific effects linked to Th2 cytokine suppression and gut-lung axis modulation. Postbiotics, including bacterial lysates (OM-85 BV, PMBL^®^), attenuated airway hyperresponsiveness and systemic inflammation. Synbiotics reduced viral respiratory infections and healthcare utilization. However, there is still a lack of direct RCTs to explore the therapeutic effects of prebiotics on pediatric asthma. Key limitations include methodological heterogeneity (dosing: 10^8^–10^10^ CFU/day; duration: 8 weeks−12 months) and risk of bias (3 low-risk, 12 with concerns).

**Conclusion:**

Our findings underscored the potential of microbiota-targeted therapies but highlight the need for standardized protocols, strain-specific trials, and pediatric prebiotic research. Future studies should integrate multi-omics to elucidate mechanisms and optimize personalized interventions.

**Systematic review registration:**

https://www.crd.york.ac.uk/PROSPERO/view/CRD42025641318, identifier: CRD42025641318.

## 1 Introduction

Asthma is a chronic inflammatory airway disease characterized by airway hyperresponsiveness, reversible airflow obstruction, and heterogeneous clinical phenotypes, affecting over 300 million individuals globally (1, 2). In children and adolescents, asthma remains the most prevalent chronic respiratory condition, according to the World Health Organization (WHO), ~14% of children worldwide experience asthma-related symptoms, with higher prevalence rates observed in high-income countries compared to low- and middle-income nations (3). Studies have shown that in 2019, the incidence of asthma in children aged 1–4 years, children aged 5–9 years, children aged 10–14 years and adolescents aged 15–19 years was 1,884.6/100,000, 980.3/100,000, 587.0/100,000, and 387.9/100,000, respectively (4). The burden of pediatric asthma is further compounded by its association with recurrent exacerbations, reduced quality of life, and significant healthcare costs (5, 6). Despite advances in pharmacotherapy, a subset of patients remains suboptimally controlled, underscoring the need for novel therapeutic strategies targeting (7).

Existing evidence highlights the gut-lung axis's multifactorial involvement in asthma pathogenesis, including immune modulation, metabolic (8–10), neuroendocrine (11, 12), and epithelial barrier mechanisms (13). Gut microbiota dysbiosis in pediatric asthma manifests not only as reduced diversity and altered *Firmicutes*/*Bacteroidetes* (14) ratio but also as disruption of microbial metabolite production, particularly short-chain fatty acids (SCFAs) like propionate and butyrate (8). These metabolites regulate pulmonary inflammation through G-protein coupled receptor-dependent pathways (9) and histone deacetylase inhibition (10), while their deficiency exacerbates Th17-mediated airway remodeling (15). Concurrently, gut barrier dysfunction permits bacterial lipopolysaccharide (LPS) translocation, activating TLR4/NF-κB signaling in alveolar macrophages and promoting neutrophilic infiltration. Notably, vagus nerve-mediated cholinergic anti-inflammatory pathways (11) and microbial-tryptophan metabolism derivatives (e.g., indole-3-aldehyde) (12) further modulate IL-22-dependent mucosal repair. Epigenetic reprogramming via microbial-derived microRNAs may additionally influence DNA methylation patterns in airway epithelial cells, predisposing to bronchial hyperresponsiveness (16). This interconnected microbial-immune-metabolic network establishes microbial homeostasis as a potential therapeutic target across asthma (17).

In recent years, various gut health interventions, including probiotics, prebiotics, synbiotics, and postbiotics, have been explored for their potential therapeutic roles in asthma management. Probiotics, such as *Lactobacillus* and *Bifidobacterium* strains, have shown immunomodulatory effects that may help reduce airway inflammation and asthma exacerbations (18). Synbiotics combining probiotics and prebiotics enhance microbial diversity and reduce wheezing episodes in high-risk infants and children (19). Postbiotics, which refer to the metabolic by-products of probiotic bacteria, may also play a role in modulating immune responses and providing protection against inflammatory conditions like asthma (20). However, existing evidence remains heterogeneous due to variations in strain specificity, dosing regimens, and outcome measures (21). A systematic synthesis of current data is critical to clarify the efficacy, safety, and mechanistic underpinnings of microbiota-targeted therapies in pediatric asthma, informing clinical guidelines and future research priorities.

## 2 Materials and methods

The research protocol has been registered with the International Prospective Register of Systematic Reviews (PROSPERO), under identifier CRD42025641318. To ensure methodological rigor and transparency, the review process strictly followed the PRISMA 2020 framework, with all checklist criteria systematically implemented to standardize reporting quality and minimize bias in evidence synthesis.

### 2.1 Sources and search strategy

The literature search was conducted from inception to 2nd January 2025 in the following electronic databases including PUBMED, EMBASE, Web of Science, and Cochrane Library. The main search string included the following words: probiotics, prebiotics, synbiotics, postbiotic, asthma and children, the detailed search strategy is provided in Supplementary material. The search was limited to human studies published in English.

### 2.2 Eligibility criteria

The inclusion criteria for eligible trials were defined as follows: (1) Trials investigating the therapeutic use of probiotics, prebiotics, synbiotics, or postbiotics in children and/or adolescents (<18 years) with asthma. (2) Studies employing a randomized controlled clinical trial design (parallel or crossover). (3) Control groups receiving placebo, usual care, conventional therapy, or no intervention. (4) Trials reporting at least one of the following outcomes: asthma exacerbation, relapse rate, symptomatic improvement (e.g., cough, wheezing), quality of life scores (validated questionnaires), Asthma Control Test (ACT) or Childhood Asthma Control Test (C-ACT), pulmonary function parameters [(e.g., the forced expiratory volume(FEV1), forced vital capacity(FVC), FEV1/FVC ratio, peak expiratory flow rate(PEFR)], inflammatory biomarkers (e.g., TNF-α, IL-10, IL-13, IFN-γ, IL-12), incidence of respiratory infections, immunological indices (e.g., IgE, IgG, CD3+ percentage, CD4+ percentage, CD4+/CD8+ ratio, NK cell activity).

The following types of studies were excluded: (1) non-human experimental models, animal studies, systematic reviews, meta-analyses, commentaries, study protocols, trial registrations, case reports. (2) Trials utilizing combined therapeutic regimens (e.g., synbiotics co-administered with vitamin D or acupuncture) that compromised intervention specificity. (3) Original full text not found.

### 2.3 Study selection and data extraction

Two investigators (D.F. and J.H.) performed duplicate independent screening of titles/abstracts followed by full-text evaluation against predefined inclusion/exclusion criteria. Discrepancies in inclusion judgments were reconciled through iterative discussion, with unresolved conflicts adjudicated by a third reviewer (N.L.). Researchers recorded the author, year of publication, country, sample size, population characteristics (age, sex, diagnosis), study design, intervention (including dosage and duration), outcome variables, and main findings from each paper, in an Excel spreadsheet.

### 2.4 Risk of bias assessment

Methodological quality appraisal was independently conducted by two investigators (D.F. and J.H.) utilizing the Cochrane RoB2 tool. Inter-rater discrepancies in bias risk classification underwent iterative adjudication by a third reviewer (N.L.) until full consensus was attained. Methodological rigor was assessed across seven domains defined by the Cochrane RoB2 framework: sequence randomization procedures, allocation concealment implementation, blinding protocols, outcome assessment objectivity, outcome data integrity, reporting transparency (selective outcome disclosure), ancillary bias sources. Domains were rated using standardized criteria (low/unclear/high risk) with explicit justification based on trial documentation.

## 3 Results

### 3.1 Literature search and selection

**Figure 1:**
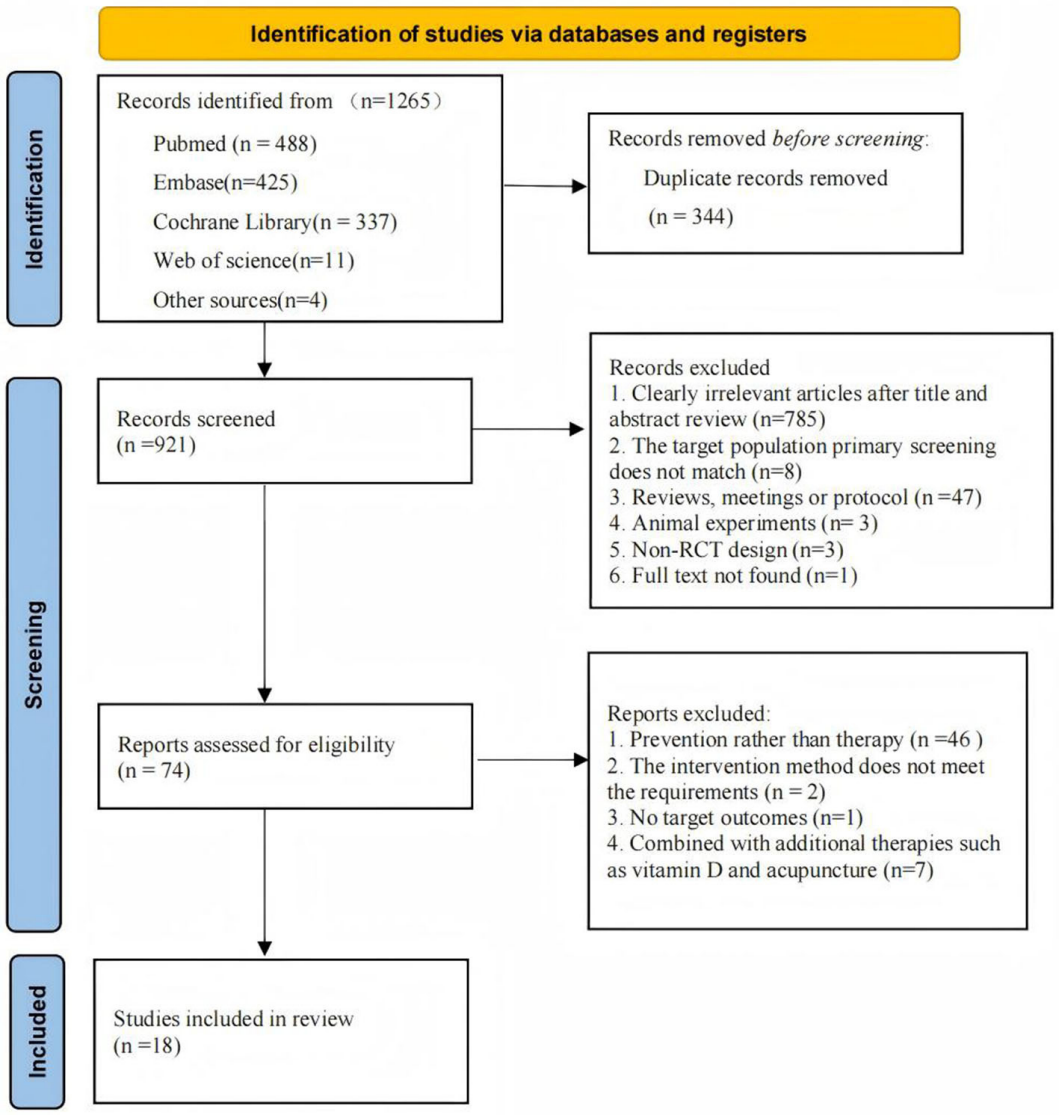
Prisma flow diagram of the selection of the articles included.

A systematic search identified 1265 potentially relevant articles, with 921 retained following duplicate removal. Title and abstract screening excluded 785 records, leaving 74 articles for full-text evaluation. Following rigorous application of inclusion criteria, 18 studies met eligibility requirements for final analysis. The study selection process is detailed in Figure 1.

### 3.2 Study characteristics

A systematic review of the 18 included investigations (22–39), comprising 13 randomized controlled trials (RCTs) and encompassing 2,419 participants, predominantly focused on pediatric populations. The majority studies exhibited a mean age range spanning childhood to preadolescence (2–11 years), only one investigation targeted infant cohorts (mean age <2 years). The male-to-female ratio approximated 1.4:1 (58.7% male, 41.3% female), reflecting a balanced yet slightly male-predominant demographic distribution. Among the 18 included studies evaluating microbial interventions: probiotics were investigated in 10 studies, with 4 deriving data from a single RCT (NCT04289441); postbiotics were assessed in 6 studies, including 2 sharing a common RCT dataset (NCT02541331); synbiotics were examined in 2 studies; no studies focused on prebiotics. Intervention durations spanned 8 weeks to 12 months. Heterogeneity in study populations included variations in asthma severity (intermittent to moderate persistent) and comorbid allergic rhinitis.

**Figure 2:**
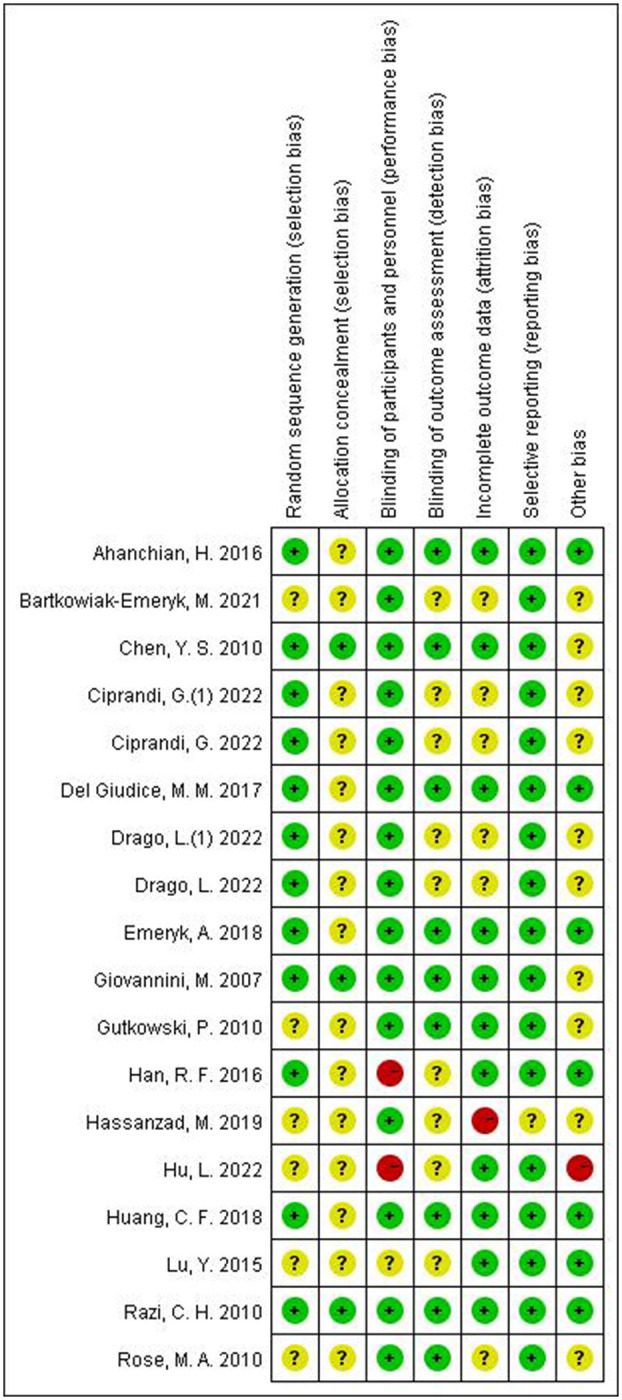
Risk assessment of bias in included studies using the tool RoB 2.0.

Risk of bias assessments for each included study are presented in Figure 2, 3 studies showed a low risk of bias, 3 studies showed a high risk of bias and 12 studies had some concerns.

### 3.3 Probiotics in the treatment of pediatric asthma

A total of 10 studies investigating the therapeutic efficacy of probiotics in pediatric asthma management were included. The studies included reports on *Lactobacillus* (*n* = 5), *Bifidobacterium* (*n* = 5), among the five studies investigating *Bifidobacterium*, four studies originated from a same RCT (NCT04289441). All of studies compared probiotics with control or placebo. Primary outcome measures comprised asthma exacerbations, pulmonary function, and immunological biomarkers. The characteristics and details of included studies were shown in Table 1.

**Table 1:** Studies of probiotics in the treatment of pediatric asthma.

**Reference**	**Country**	**Sample size (M/F)**	**Population**	**Age in intervention group**	**Age in control group**	**RCT design (blinding)**	**Duration**	**Intervention of experimental group**	**Intervention of control group**	**Outcomes**	**Main Finding**
Giovannini et al. (22)	Italy	187(120/67)	Pre-School Children With llergic Asthma and/or Rhinitis	2–5 years	2–5 years	Parallel (yes)	12 months	Fermented milk (100 mL/day) with *L. casei DN-114 001* (10^8^ CFU/mL), yogurt cultures (*L. bulgaricus* and *S. thermophilus*). *n =* 62	Non-fermented milk (100 mL/day) *n =* 57	Time free from asthma/rhinitis episodes; number/duration of episodes Fever/diarrhea episodes Immunological profile (IgA, IgE, IgM, IgG) adverse events (fever, diarrhea)	↓ Annual rhinitis episodes No significant difference in asthma episodes or duration. ↓ duration of diarrhea episodes No significant changes in serum IgA, IgG, IgM, or IgE levels.
Chen et al. (23)	China	105(60/55)	Asthmatic Children With Allergic Rhinitis	8.1 ± 3.0 years	9.4 ± 4.1 years	Parallel (yes)	8 weeks	Probiotic: *L. gasseri A5* (2 × 10^9^ CFU/capsule), twice daily. *n =* 49	Placebo *n =* 56	Pulmonary Function Tes(FEV1, FVC, FEV1/FVC(%), PEFR and MEF25–75) Asthma and AR Symptoms scores Severity of Asthma and AR Asthma Control Test Results inflammatory cytokine (TNF-a, IL-10, IL-13,INF-γ, IL-12) Serum IgE	↑ FEV1, FVC, and PEFR ↓ Daytime asthma and AR symptom scores ↓TNF-α, IFN-γ, IL-12, and IL-13 production by PBMCs
Gutkowski et al. (24)	Poland	46 (26/20)	Mild to moderate atopic asthma	6.93 (4.3–9.9) years	6.65 (4.2–9.7) years	Parallel (yes)	12 weeks	1.6 × 10^9^ lactic acid bacteria per capsule (*L. acidophilus* 37.5%, *B. bifidum* 37.5%, *L. delbrueckii subsp. bulgaricus* 25%); administered twice daily (total daily dose: 3.2 × 10^9^ CFU) *n =* 22	Placebo *n =* 24	Bronchodilators used Asthma exacerbations, FEV1 %VC, HLA DR %, CD8/CD45RA %	↑ FEV1%VC ↓asthma exacerbations. ↓bronchodilator use. ↑ HLA-DR expression on monocytes, ↓ CD8+CD45RA+ lymphocytes.
Del Giudice et al. (30)	Italy	40(18/22)	children with seasonal allergic rhinitis and intermittent asthma	9 ± 2.2 years	9 ± 2.2 years	Parallel (yes)	8 weeks	oral supplementation containing *Bifidobacteria* mixture, *B..longum BB536* 3 × 10^9^CFU), *B. infantis M-63* (1 × 10^9^CFU), and *B. breve M-16 V* (1 × 10^9^ CFU) as powder in 3 mg sachet per day *n =* 20	Placebo *n =* 20	Nasal symptoms scores Quality of life scores Rescue medication use	↓ AR symptoms ↑ QoL No difference in rescue medication use.
Huang et al. (32)	China	147(83/64)	Intermittent to moderate persistent asthma	7.36 ± 2.13 years	7.86 ± 2.50 years	Parallel (yes)	3 months	LP: *L. paracasei GMNL-133* (3 × 10^9^ CFU/day) LF: *L. fermentum GM-090* (3 × 10^9^ CFU/day) LP+LF: Combination of LP and LF doses *n =* 112	Placebo *n =* 35	C-ACT scores PAQLQ scores PASSs, PEFRs medication use Skin prick IgE,IFN-γ, IL-4, and TNF-α levels	↓ asthma severity ↑ C-ACT scores ↑ PEFR and ↓ IgE levels
Ciprandi et al. (35)	Italy	160(92/68)	Preschoolers with persistent wheezing	4.3 ± 0.78 years	4.4 ± 0.78 years	Parallel (yes)	4 months	Probiotic mixture: *B. breve B632* (≥1 × 10^9^ CFU) + *Ligilactobacillus salivarius LS01* (≥1 × 10^9^ CFU), twice daily for 8 weeks, then once daily for 8 weeks. *n =* 89	Placebo *n =* 71	Number of children with wheezing episodes	↓ wheezing episodes ↓ inhaled corticosteroid use: No difference in oral corticosteroid use
Ciprandi et al. (36)	Italy	164 (104/60)	Asthmatic children with Allerg	8.8 ± 3.31 years	8.8 ± 3.31 years	Parallel (yes)	4 months	Probiotic mixture: *B. breve B632* (≥1 × 10^9^ CFU) + *Ligilactobacillus salivarius LS01* (≥1 × 10^9^ CFU), twice daily for 8 weeks, then once daily for 8 weeks. *n =* 80	Placebo *n =* 84	Number of Asthma Exacerbations	↓ asthma exacerbations
Drago et al. (37)	Italy	422(240/182)	Children with asthma	7.0 ± 3.38 years	7.0 ± 2.95years	Parallel (yes)	4 months	Probiotic mixture: *B. breve B632* (≥1 × 10^9^ CFU) + *Ligilactobacillus salivarius LS01* (≥1 × 10^9^ CFU), twice daily for 8 weeks, then once daily for 8 weeks. *n =* 212	Placebo *n =* 210	Number of Asthma Exacerbations Severity and duration of exacerbations during the study Maintenance therapy over time: intensity of asthma treatment medication use	↓ asthma exacerbations
Drago et al. (37)	Italy	262(148/114)	Schoolchildren with asthma	9.0 ± 3.18 years	8.4 ± 2.72 years	Parallel (yes)	4 months	Probiotic mixture: *B. breve B632* (≥1 × 10^9^ CFU) + *Ligilactobacillus salivarius LS01* (≥1 × 10^9^ CFU), twice daily for 8 weeks, then once daily for 8 weeks. *n =* 123	Placebo *n =* 139	Number of Asthma Exacerbations Severity of exacerbations	↓ asthma exacerbations
Rose et al. (26)	Germany	131 (80/51)	Infants aged 6–24 months with recurrent wheezing and family history of atopy	16.7 ± 5.52 months	14.4 ± 5.83 months	Parallel (yes)	6 months	*L. rhamnosus* (LGG, 1 × 10^10^ CFU) twice daily *n =* 65	Placebo *n =* 66	Clinical outcome parameters (SCORAD indices, Number of wheezing episodes, Days with wheeze...) IgE, Eosinophils (%), eosinophilic cationic protein,	No clinical improvement ↓ aeroallergen sensitization: Lower cumulative aeroallergen-specific IgE

*L., Lactobacillus; B., Bifidobacterium; C-ACT, Childhood-Asthma Control Test; PAQLQ, Pediatric Asthma Quality of Life Questionnaire; PASSs, Pediatric Asthma Severity Scores; PEFRs, peak expiratory flow rates; QoL, Quality of life; SCORAD, the severity scoring of atopic dermatitis*.

#### 3.3.1 Asthma exacerbation

Seven studies (22, 24, 26, 35–38) evaluated the effects of probiotics on asthma exacerbations. Five of these investigations demonstrated significant reductions in asthma exacerbations following probiotic administration. One study reported a markedly lower incidence of asthma exacerbations in the probiotic group (9%) compared to placebo (23.8%), with an odds ratio (OR) of 3.17 (*p* < 0.001) (37). Conversely, one study observed no statistically significant difference in asthma episode frequency or duration between intervention and control groups (*P* > 0.05) (22). Furthermore, a longitudinal analysis evaluating exacerbation patterns showed no statistically significant differences in wheezing episodes or days with wheeze during the 6-month intervention period and the subsequent 6-month follow-up phase (*P* > 0.05) (26).

#### 3.3.2 Pulmonary function

Among all included studies on probiotic interventions, three investigated pulmonary function outcomes (23, 24, 32). Pooled data of these trials demonstrated significant improvements in pulmonary function indices within the probiotic intervention groups. Specifically, one trial documented enhanced lung function parameters [FEV1, FVC, and FEV1/FVC (%)] in the probiotic cohort compared to the control group (*P* < 0.05) (23). Another study showed improved PEFR in *Lactobacillus paracasei GMNL-133* + *Lactobacillus fermentum GM-090* group (32).

#### 3.3.3 C-ACT scores

Two randomized trials evaluated the impact of probiotics vs. placebo on Childhood Asthma Control Test (C-ACT) scores. Both studies established significant C-ACT score enhancements with probiotic supplementation. Specifically, One study reported a significantly higher proportion of patients achieving improvements in C-ACT scores within the probiotic cohort (67.3%, 33/49) compared to the control group (58.9%, 33/56; *P* < 0.05) (23). Another study employed generalized estimating equations (GEE) with age-sex adjustments, demonstrating clinically relevant C-ACT improvements across probiotic arms (*L. paracasei* [LP], *L. fermentum* [LF], and LP+LF) (*P* < 0.05) (32).

#### 3.3.4 Immune biomarker levels

Among the included studies, five investigated immune biomarker profiles, including IgE, IgG, TNF-α, IL-10, IL-13, IFN-γ, and IL-12. Regarding IgE-specific parameters, one trial documented significantly reduced total serum IgE levels in the *L. paracasei* (LP) + *Lactobacillus fermentum* (LF) intervention group post-treatment (*P* < 0.05) (32), while another study observed decreased cumulative aeroallergen-specific IgE in the probiotic cohort compared to controls (*P* = 0.027 post-intervention; *P* = 0.03 at follow-up) (26). In contrast, two other trials found no statistically significant intergroup differences in IgE levels (22, 23). For inflammatory mediators, PBMC analysis in one study revealed significant reductions in TNF-α, IFN-γ, IL-12, and IL-13 production following probiotic administration (23). Conversely, serum concentrations of IFN-γ, IL-4, and TNF-α remained unchanged in another investigation (32).

### 3.4 Postbiotics in the treatment of pediatric asthma

The efficacy of postbiotics was also explored in six studies (25, 27, 29, 31, 34, 39). The intervention used in the six studies was bacterial lysate at a dose of 3.5–7mg/day. All of studies compared bacterial lysate with conventional treatment or placebo. Primary outcome measures comprised asthma exacerbations, acute respiratory tract illnesses (ARTIs), pulmonary function, and immunological biomarkers. The details of included studies were shown in Table 2.

**Table 2:** Studies of postbiotics in the treatment of pediatric asthma.

**Reference**	**Country**	**Sample size (M/F)**	**Population**	**Age in intervention group**	**Age in control group**	**RCT design (blinding)**	**Duration**	**Intervention of experimental group**	**Intervention of control group**	**Outcomes**	**Main finding**
Razi et al. (25)	Turkey	75(55/20)	preschool children with recurrent wheezing	26 (16–37) months	24.5 (14–45) months	Parallel (yes)	3 months	OM-85 BV (3.5 mg/d) for 10 consecutive days in each of 3 months *n =* 35	Placebo *n =* 40	number of wheezing attacks number of ARTIs number of acute nasopharyngitis efficacy parameters (hospitalizations, steroid therapy)	↓wheezing attacks ↓ARTIs ↓ duration of wheezing attacks ↓acute nasopharyngitis
Lu et al. (27)	China	60(38/22)	Children diagnosed with asthma	8.9 ± 2.8 years	8.7 ± 2.7 years	Parallel (yes)	9 months	OM-85 BV (3.5 mg/d for patients 5–12 years old or of 7.0 mg/d for those >12 years old) for a total of 10 days per month over 3 months. Treatment was withheld for the remaining 20 days of each month. Treatment consisted of two 3-month courses in a 9-month period, taking place from the 1st to the 3rd month and the 7th to the 9th month. *n =* 24	Conventional therapy *n =* 36	Respiratory infections Asthma attacks IFN -γ, IL-4, and IL-10 NKT and CD4+ NKT cell percentages in peripheral blood clinical effects (Cough, Wheezing, Antibiotics use)	↓respiratory infections ↓asthma attacks ↑NKT/CD4+ NKT cells ↑ IFN-γ/IL-4 ratio, ↑ IL-10
Han et al. (29)	China	136(71/65)	Capillary bronchitis secondary bronchial asthma	2.3 ± 0.6 years	2.2 ± 0.4 years	Parallel (not specified)	3 months of intervention 1-year follow-up	Broncho-Vaxom OM-85BV (3.5 mg/d) for 10 consecutive days in each of 3 months *n =* 74	Conventional therapy *n =* 62	Onset Frequency and Duration of Capillary Bronchitis and Asthma IL-17, IL-4, IL-10 and IFN-γ α7nAChR expression level (%)	↓frequency and duration of capillary bronchitis and asthma attacks ↓ IL-17, IL-4; ↑ IL-10, IFN-γ. ↓α7nAChR expression in CD4+ T cells
Emeryk et al. (31)	Poland	150 (74/76)	Children with asthma	9.3 ±2.7 years	9.8 ±2.6 years	Parallel (yes)	3 months	PMBL^®^ Tablet contains (7 mg/d of bacterial lysate) for the first 10 days of each month, for three consecutive months *n =* 74	Placebo *n =* 76	C-ACT/ACT scores number of respiratory tract infections the number of asthma exacerbations times to first, second and third exacerbation number of days of SABA use for relief of exacerbation over the whole study period	↓ asthma exacerbations ↓ infections leading to exacerbations ↑ asthma control scores (C-ACT/ACT changes).
Bartkowiak-Emeryk et al. (34)	Poland	49(39/10)	Hildren with allergic asthma and house dust mites allergy	10.67 ± 2.13 years	9.54 ± 2.52 years	Parallel (yes)	3 months	PMBL^®^ Tablet (7 mg/d of bacterial lysate) for the first 10 days of each month, for three consecutive months *n =* 21	Placebo *n =* 28	Blood cells count lymphocytes	↑ T lymphocytes (CD3+), Treg (CD4+CD25+FOXP3+), Tc (CD8+), and NK cells (CD3–CD16+CD56+). ↓ activated T cells (CD25+/CD69+). ↑ immune modulation in asthma.
Hu et al. (39)	China	132(78/54)	Children with Acute Bronchial Asthma	10.89 ± 0.31 years	10.23 ± 0.24 years	Parallel (yes)	3 months	Conventional treatment + bacterial lysate capsules (3.5 mg/d) orally, 10 days/month × 3 months). *n =* 66	Conventional treatment *n =* 66	Clinical Efficacy Symptom Improvement Time Pulmonary Function [FEV1(L),PEF(L/s), FVC(L),MEF(L/s)] Serum GAL-3, ECP And SAA Levels Immune Function Indexes [CD3+ (%), CD4+ (%),CD4+/CD8+, NK (%)]	↑ clinical efficacy ↑ Faster symptom improvement ↑ pulmonary function: Higher FEV1, PEF, FVC, and MEF ↓ serum Gal-3, ECP, and SAA levels ↑ CD3+, CD4+, CD4+/CD8+, and NK cells

*ARTIs, acute respiratory tract illnesses; SABA, short-acting* β*2-agonists; C-ACT, Childhood-Asthma Control Test; ACT, Asthma control Test*.

#### 3.4.1 Asthma attacks

Four studies (25, 27, 29, 31) assessed the therapeutic impact of postbiotics on asthma exacerbations, with all trials demonstrating the efficacy of bacterial lysates in reducing attack frequency. Notably, one randomized controlled trial (RCT) observed a 37.9% decrease in wheezing episodes with OM-85 BV vs. placebo (*P* < 0.001) (25). Another study revealed superior asthma control through bacterial lysate administration compared to standard inhaled corticosteroids (ICS), evidenced by significantly fewer exacerbations (0.9 vs. 1.8 attacks; *P* = 0.01) in the OM-85 BV cohort (27). Furthermore, a comparative study documented enhanced clinical efficacy when bacterial lysate was combined with ICS (95.45% vs. 84.85% improvement rate; *P* < 0.05) (39).

#### 3.4.2 ARTIs

Among all included studies, three trials evaluated bacterial lysate interventions for reducing acute respiratory tract infections (ARTIs), with all demonstrating significant efficacy. A RCT documented a 31.4% reduction in ARTI incidence with OM-85 BV vs. placebo (*P* < 0.001) (25). Another comparative study reported lower respiratory infection rates in the OM-85 BV cohort compared to conventional inhaled corticosteroid therapy (mean episodes: 3.1 vs. 7.4; *P* < 0.01) (27). Furthermore, a third study revealed fewer asthma-exacerbating infections in the PMBL^®^ group relative to controls (10 vs. 21 cases; *P* = 0.002) (31).

#### 3.4.3 Immune biomarker levels

Among the included studies, four investigated immune biomarker profiles, with all demonstrating the efficacy of bacterial lysates in enhancing immunological parameters. Regarding immune cell modulation: one study reported increased NKT/CD4+ NKT cells in experimental group (*P* < 0.01) (27), one study reported increased T lymphocytes (CD3+), Treg (CD4+CD25+FOXP3+), Tc (CD8+), and NK cells (CD3-CD16+CD56+), and decreased activated T cells (CD25+/CD69+) in experimental group (34). Hu et al. (39) showed increased CD3+, CD4+, CD4+/CD8+, and NK cells in experimental group (*P* < 0.001). Regarding inflammatory markers, Lu et al. (27) demonstrated increased IFN-γ/IL-4 ratio, IL-10 in OM-85 BV group (*P* < 0.01). Han et al. (29) confirmed that IL-17and IL-4 decreased in the experimental group, while IL-10 and IFN-γ increased.

#### 3.4.4 Pulmonary function

Only one study, Hu et al. (39), reported the effect of bacterial lysate on pulmonary function. The study showed improved pulmonary function: Higher FEV1, PEF, FVC, and MEF in the bacterial lysate plus conventional inhaled corticosteroid compared with conventional treatment (*P* < 0.001).

### 3.5 Synbiotics in the treatment of pediatric asthma

As shown in Table 3, a total of 2 studies investigating the therapeutic efficacy of synbiotics in pediatric asthma management were included. The two studies used interventions Lactocare^®^ and Kidilact^®^, respectively, with placebo in the control group. Ahanchian et al. (28) reported reduced viral respiratory infections (0.44 vs. 0.74 episodes; *P* = 0.007) and lower salbutamol use in the synbiotics group (*P* = 0.017). Hassanzad et al. (33) reported reduced outpatient visits (19 vs. 55; *P* = 0.001) and higher satisfaction in the synbiotics group (78.3% vs. 25.7%), while no significant difference in asthma attacks or hospitalizations.

**Table 3:** Studies of synbiotics in the treatment of pediatric asthma.

**Reference**	**Country**	**Sample size (M/F)**	**Population**	**Age in intervention group**	**Age in control group**	**RCT design (blinding)**	**Duration**	**Intervention of experimental group**	**Intervention of control group**	**Outcomes**	**Main finding**
Ahanchian et al. (28)	Iran	72 (45/27)	Mild persistent asthma,	8.1 ± 1.7 years	8.2 ± 2.1 years	Parallel (yes)	2 months	Lactocare^®^, a Synbiotic containing 1 billion CFU/Capsule of *L. casei, L. rhamnosus, Streptococcus thermophilus,B. breve, L.s acidophilus, B. infantis, L. bulgaricus*, and Fructooligosacharide daily *n =* 36	Placebo *n =* 36	Number of respiratory viral infection episodes Acute viral upper respiratory tract infection episodes drug use Outpatient visits, School absent, Hospital admission	↓viral respiratory infections in the first month ↓ salbutamol use
Hassanzad et al. (33)	Iran	81(48/33)	Children with asthma	6.9 ± 2.7 years	6.6 ±2.4 years	Parallel (yes)	6 months	Kidilact^®^ (*L. Casei, B. infantis,L. acidophilus B. breve, L. rhamnosus Streptococcus, thermophiles, L. bulgaris*, Fructooligosaccharide) once a day *n =* 46	Placebo *n =* 35	Frequency of asthma attacks visits to a hospital due to respiratory complaints or exacerbated the rate of hospitalization due to asthma attacks participants' level of satisfaction from their treatment and the side effects of the medications	↓ outpatient visits No significant difference in asthma attacks or hospitalizations. ↑satisfaction in the experimental group

*L., Lactobacillus; B., Bifidobacterium*.

### 3.6 Safety and adverse events

Across all studies, probiotics, postbiotics, and synbiotics were generally well-tolerated. Most studies reported no severe adverse effects. Mild and transient adverse events, such as abdominal symptoms and fever, were noted in some trials (22, 25, 27, 33) but occurred at similar rates in both intervention and control groups.

## 4 Discussion

This systematic review synthesizes evidence from 18 randomized controlled trials (RCTs) to evaluate the efficacy and safety of microbiome-targeted interventions—probiotics, synbiotics, postbiotics, and prebiotics—in pediatric asthma management. By adhering to PRISMA guidelines, we analyzed 2,419 participants aged <18 years, with a focus on clinically relevant endpoints such as asthma exacerbations, pulmonary function, and immune modulation. The included studies predominantly investigated *Lactobacillus* and *Bifidobacterium* strains (10 RCTs), bacterial lysates (6 RCTs), and synbiotic formulations (2 RCTs), while identifying a critical gap in prebiotics research.

### 4.1 Effect of probiotics, prebiotics, synbiotics and postbiotics on pediatric asthma

The current synthesis underscores the potential of probiotics in modulating asthma outcomes, albeit with notable heterogeneity across studies. Overall, probiotics demonstrated efficacy in reducing asthma exacerbations (35, 37), improving pulmonary function (32), and modulating immune profiles (23). However, heterogeneity persisted across studies: *Lactobacillus* monotherapy (e.g., *L. gasseri A5* in Chen et al. (23)) improved lung function but had limited impact on asthma exacerbations, while *Bifidobacterium* strains (e.g., *B. breve B632* in Ciprandi et al. (35)) reduced inhaled corticosteroid use. Multi-strain formulations, such as *L. paracasei* + *L. fermentum* in Huang et al. (32), showed synergistic benefits, enhancing PEFR improvement and IgE reduction effects compared to single strains. Emerging evidence suggests that the therapeutic effect of probiotics in pediatric asthma involves multifactorial pathways. Probiotics, particularly *Lactobacillus* and *Bifidobacterium* strains, restore the Th1/Th2 balance by suppressing Th2 cytokines (IL-4, IL-5, IL-13) and enhancing Th1 cytokines (IFN-γ), thereby counteracting allergic inflammation (40–42). Concurrently, they induce regulatory T cells (Tregs) to secrete anti-inflammatory IL-10 and TGF-β, dampening airway hyperresponsiveness (43, 44). Study shown that *Bifidobacterium*-derived acetate and propionate likely enhance Treg differentiation, suppressing eosinophilic inflammation (8), while *Lactobacillus* strains modulate dendritic cell tolerance, reducing allergen-specific IgE (45). By modulating gut microbiota, probiotics promote the production of metabolites such as short-chain fatty acids (SCFAs) and tryptophan. SCFAs inhibits histone deacetylases (HDACs), reducing airway inflammation epigenetically (46). Tryptophan metabolites (e.g., Indole-3-Lactic Acid) suppress TNF-α and IL-6 release via AhR signaling in alveolar macrophages (47, 48). Additionally, probiotics strengthen mucosal barriers in the gut and lungs via upregulation of tight-junction proteins (occludin, claudin), limiting allergen/pathogen translocation (49–51). Probiotics prime airway epithelial cells to upregulate pattern-recognition receptors and boost type I interferons (IFN-α/β), enhancing antiviral responses and reducing viral-triggered exacerbations (52).

Bacterial lysates (e.g., OM-85 BV and PMBL^®^), classified here as postbiotics, demonstrated consistent efficacy in reducing asthma attacks and ARTIs across six studies (25, 27, 29, 31, 34, 39). Their immunomodulatory effects, including enhanced NK cell activity and IFN-γ/IL-4 ratio shifts (27). Mechanistically, these lysates contain microbial components (e.g., peptidoglycans, lipoteichoic acids) that act as pathogen-associated molecular patterns (PAMPs), engaging Toll-like receptors (TLRs) and NOD-like receptors (NLRs) to activate innate immunity (53). TLR2/4 signaling primes dendritic cells to promote a Th1-skewed response, evidenced by elevated IFN-γ/IL-4 ratio (27), while suppressing eosinophilic inflammation via IL-10 upregulation (29). This aligns with the paradigm of microbial training of innate immunity, where bacterial lysates induce epigenetic reprogramming in epithelial cells, enhancing antiviral defense (54).

Limited evidence from two RCTs suggests that synbiotics interventions may reduce healthcare utilization in pediatric asthma, including decreased outpatient visits (33), and attenuate viral respiratory infections (28). Notably, however, one study found no significant reduction in asthma exacerbation frequency with synbiotics, underscoring the need for large-scale RCTs to validate their therapeutic role in childhood asthma (33). Moreover, both studies exhibit methodological limitations in intervention details. The original trial failed to explicitly report quantitative dosage parameters of the synbiotic formulation, including CFU counts per strain and precise prebiotic content, which may affect the reproducibility of the findings and comparability with other clinical studies. Synbiotics—combinations of probiotics (live beneficial microorganisms) and prebiotics (non-digestible substrates that selectively promote microbial growth)—leverage synergistic interactions to enhance gut microbiota modulation. Prebiotics such as fructooligosaccharides (FOS) and galactooligosaccharides (GOS) act as fermentable substrates for probiotic strains (e.g., *Bifidobacterium* and *Lactobacillus*), improving their survival, colonization, and metabolic activity in the gut lumen. This synergy may theoretically strengthen mucosal immunity by promoting microbial diversity and metabolic output (55). Contrasting these mechanistic insights, a 3-arm crossover RCT involving 17 patients with stable asthma demonstrated that inulin alone led to significant improvements in the Asthma Control Questionnaire scores, reduced sputum eosinophil percentage, and downregulated sputum histone deacetylase 9 mRNA expression. Intriguingly, these benefits were absent in the combined inulin-probiotic group (56). Consequently, whether probiotics and prebiotics exert additive or synergistic effects in asthma management remains unresolved, necessitating further rigorously designed trials.

This review included 0 studies on prebiotics in the treatment of childhood asthma. Emerging evidence from adult asthma RCTs suggests potential benefits of prebiotics. In adults, interventions with galactooligosaccharides (GOS) and inulin have demonstrated improving asthma. In an RCT, adults with asthma receiving 5.5 g/day Bimuno-galactooligosaccharide (B-GOS) for 3 weeks exhibited a 40% attenuation in post-hyperpnoea FEV1 decline alongside reduced systemic inflammation markers, including TNF-α and CCL17, demonstrating prebiotic modulation of airway hyperresponsiveness and inflammation in asthma (57). Another trial using soluble fiber (inulin 12 g/day) in adults with stable asthma reported improvements in airway inflammation, asthma control and gut microbiome composition (56). Observational data demonstrated a link between low dietary fiber intake and pediatric asthma severity (58). Current RCTs on prebiotics in children primarily focus on evaluating the preventive effects of prebiotic supplementation against allergies, atopic dermatitis, and respiratory infections in healthy children or those at high risk of atopy (59–63). However, evidence on the therapeutic efficacy of prebiotics in children with asthma remains scarce, highlighting a critical gap in current research.

### 4.2 Implications for practice

Included studies in this review have corroborated the beneficial effects of probiotics and synbiotics in the treatment of pediatric asthma, thereby emphasizing their potential as adjunctive therapeutic modalities. Notwithstanding, a notable limitation in current research is the scarcity of direct comparisons among different strains. This scarcity makes it challenging for clinicians to make informed decisions regarding the most effective strain or combination of strains for individual patients. Furthermore, the substantial variability in dosing (ranging from 10^8^ to 10^10^ CFU/day) and intervention durations (spanning from 8 weeks to 12 months) of probiotics across studies accentuates the exigency for standardized guidelines. Pending the establishment of such guidelines for prebiotic use in children with asthma, clinicians could determine their medication regimen based on other pediatric probiotic guidelines (64–67), as well as available evidence, and closely monitor patient efficacy and safety.

Bacterial lysates, which have demonstrated efficacy in ameliorating asthma symptoms, reducing acute respiratory tract illnesses, and enhancing immunity, presents a viable option for clinicians. Therapies based on bacterial lysates, such as OM-85 BV or PMBL^®^, could be considered for incorporation into treatment plans, particularly for patients with recurrent exacerbations.

Although probiotics, synbiotics, and postbiotics generally manifested favorable safety profiles in the studies included in this review, mild and transient adverse events were nonetheless reported. Clinicians should remain vigilant in monitoring patients for any adverse effects, including abdominal discomfort, rash, and fever, especially within high-risk subgroups such as immunocompromised children.

In summary, while microbiota—targeted interventions hold great promise for the management of pediatric asthma, the current body of evidence is circumscribed by research gaps. Clinicians should exercise caution, stay updated on emerging research, and consider referring patients to well—designed clinical trials to further advance the field.

### 4.3 Implications for research

Based on some limitations of the studies included in this paper, suggestions for future research directions are proposed to promote the development of the field of microbiome targeted intervention in pediatric asthma.

Research on probiotics for the treatment of childhood asthma has the following limitations. Firstly, *Bifidobacterium* strains from a single RCT dominated the evidence base (NCT04289441), which limited generalizability and highlighted the need for diversification in probiotic research. Secondly, heterogeneous dosing (10^8^ - 10^10^ CFU/day), intervention durations (8 weeks−12 months) and outcome indexs failed to standardize the optimal optimal protocol and reduced comparability, which echoing concerns raised by Cuello-Garcia et al. (68). Thirdly, 90% of studies focused on *Lactobacillus* and *Bifidobacterium*, neglecting other emerging bacteria that may have immunomodulatory effects. Future researches should prioritize head-to-head comparative studies of single- vs. multi-strain formulations and distinct strains, explore novel microbial species, and integrate multi-omics approaches to optimize therapeutic strategies.

Current researches on postbiotics for pediatric asthma treatment primarily utilizes bacterial lysates. However, the classification of bacterial lysates as postbiotics remains contentious, with ISAPP defining postbiotics as “preparation of inanimate microorganisms and/or their components that confers a health benefit on the host”, and excluding bacterial lysates from postbiotics classification (69). While there are still numerous contemporary studies continue to categorize bacterial lysates as postbiotics (20, 70, 71). Despite this ambiguity, the bacterial lysates incorporated in the our study exemplify the inanimate nature and health benefit properties characteristic of postbiotics. Further standardization of postbiotic definitions is critical to harmonize research and clinical applications. Standardizing the definition would enable more accurate comparison of studies and facilitate the development of evidence—based guidelines for the use of postbiotics in pediatric asthma treatment.

Existing research frameworks lack robust evidence regarding the clinical therapeutic value of prebiotic formulations in children with diagnosed asthma. This gap highlights the urgent need for pediatric trials to validate prebiotics as a viable therapeutic strategy inpediatric asthma. Future research should prioritize the design and implementation of well—controlled pediatric RCTs of therapeutic efficacy of prebiotics in children with asthma.

Regarding synbiotics, there remains a pressing need for more standardized and large-scale clinical trials to verify their effects on the treatment of childhood asthma. These trials should assess multiple endpoints, including asthma exacerbation, pulmonary function improvement, immunological biomarker modulation, and quality of life.

### 4.4 Strengths and limitations

This review represents the first systematic synthesis of evidence on the therapeutic roles of probiotics, synbiotics, prebiotics, and postbiotics in pediatric asthma. Its novelty and comprehensiveness provide a critical foundation for guiding clinical practice and future research directions in microbiome-targeted interventions for pediatric asthma.

However, our study is subject to several limitations mainly stemming from the current state of research in this field. Firstly, heterogeneity in outcome measures across studies was unavoidable, such as asthma remission rates, exacerbation frequency, number of exacerbation and symptom scores, which precluded quantitative meta-analysis, restricting conclusions to qualitative synthesis. Secondly, while the inclusion criteria targeted individuals aged <18 years, the enrolled population predominantly comprised children aged 2–11 years, with only one study including infants and toddlers (<2 years). Despite initial plans to perform subgroup analyses to differentiate effects across developmental stages (infants, children, and adolescents), the limited age diversity hindered planned subgroup analyses to assess age-specific effects. Thirdly, the heterogeneity including strain specificity, dosage variability and intervention durations among observed probiotic or synbiotic formulations complicated the identification of optimal regimen, resulting in uncertainty in formulating clinical recommendations. Lastly, included studies were predominantly conducted in Europe (Italy, Poland, Germany) and Asia (China, Iran, Turkey), with no representation from North/South America, Africa, or Oceania. This regional skew may limit the generalizability of findings, as genetic, environmental, and socioeconomic factors influencing asthma and gut microbiota vary globally.

## 5 Conclusion

This systematic review synthesizes emerging evidence supporting the therapeutic potential of probiotics, postbiotics, and synbiotics in pediatric asthma management. Probiotic interventions, particularly *Lactobacillus* and *Bifidobacterium* strains, demonstrate efficacy in reducing asthma exacerbations and improving pulmonary function, likely through Th1/Th2 immune balance restoration and gut-lung axis modulation. Postbiotics, such as bacterial lysates, show promise in reducing airway hyperresponsiveness and systemic inflammation, though their classification remains debated. Synbiotics further enhance clinical outcomes by reducing viral respiratory infections and healthcare utilization. However, significant heterogeneity in strain selection, dosing, and outcome measures, coupled with methodological limitations, constrain definitive conclusions. Notably, there is a conspicuous absence of direct RCTs dedicated to exploring the therapeutic effects of prebiotics in pediatric asthma. Future research could prioritize strain-specific trials, standardized protocols, and mechanistic studies integrating multi-omics to validate gut microbiota-targeted therapies.

## Data Availability

The original contributions presented in the study are included in the article/Supplementary material, further inquiries can be directed to the corresponding author.
