# Supplementary material for: Effects of probiotics, prebiotics, synbiotics and postbiotics on pediatric asthma: a systematic review
Source: Front Nutr. 2025 Apr 25;12:1586129. doi: 10.3389/fnut.2025.1586129 (PMC12061971; doi:10.3389/fnut.2025.1586129)
Supplement: Supplementary file 1 [file Table_1.docx]

**Effects of probiotics, prebiotics, synbiotics and postbiotics on pediatric asthma: a systematic review**

Appendix Table 1 Summary of the PICOS criteria used to identify studies to be included

| **Parameter** | **Description** |
| --- | --- |
| Population | Children or adolescents (＜18 years) with asthma |
| Intervention | Eligible interventions include any probiotics, prebiotics synbiotics (e.g., products that contain probiotics and prebiotics ) and postbiotics |
| comparison | Placebo, usual care, conventional therapy，or no intervention |
| Outcomes | asthma exacerbation, relapse rate, symptomatic improvement, quality of life scores (validated questionnaires), Asthma Control Test (ACT) or Childhood Asthma Control Test (C-ACT), pulmonary function parameters, inflammatory biomarkers , incidence of respiratory infections, immunological indices |
| Design | Randomized controlled trial (parallel or cross-over designs.) |

**Probiotics** – defined in this review as living bacteria (the internationally endorsed definition is live microorganisms that, when administered in adequate amounts, confer a health benefit on the host)

**Prebiotics** – defined as non-digestible compounds that provides a beneficial effect on the host by stimulating the growth of selected indigenous bacteria including inulin, galacto-, fructo-, human milk-oligosaccharides

**Postbiotics -**a preparation of inanimate microorganisms and/or their components that confers a health benefit on the host

Appendix Table 2 Search strategy (Date 2025-1-2)

| **PubMed database** | | |
| --- | --- | --- |
| Search number | Search Details | Results |
| 1 | "probiotics"[Mesh] OR probiotic* OR Saccharomyce* OR Bifidobacteri* OR Lactobacill* OR Streptococc* OR Enterococc*  OR Lactococc* OR Bacillus bacterium OR Escherichia coli OR Bacillus OR Clostridium OR Enterococcus faecalis OR Propionibacterium | 984442 |
| 2 | "prebiotics"[Mesh] OR prebiotic* OR inulin OR galactose oligosaccharide OR galactose oligomer OR oligogalactose OR fructan* OR  fructooligosaccharide* OR fructo-oligosaccharide* OR oligofructose OR galactooligosaccharide* OR  galacto-oligosaccharide* OR oligosaccharide* OR Idolax OR Raftilose P95 | 82501 |
| 3 | "synbiotics" [Mesh] OR synbioti* | 3181 |
| 4 | Postbiotic* OR "bacterial lysates" [Mesh] OR Paraprobiotics OR Non-viable probiotics OR Heat-killed probiotics OR Tyndallized probiotics | 1398 |
| 5 | "asthma"[MeSH] OR asthma* OR wheeze* OR wheezing* OR respiratory allergy* OR Bronchial Asthma | 260533 |
| 6 | "Child"[MeSH] OR child* OR "Adolescent"[MeSH] OR adolescen* OR teens OR teen OR teenager* OR childhood OR youth* OR juvenile* OR minors | 5072820 |
| 7 | (randomized controlled trial[pt] OR controlled clinical trial[pt] OR randomized[tiab] OR placebo[tiab] OR drug therapy[sh] OR randomly[tiab] OR trial[tiab] OR groups[tiab]) NOT (animals[mh] NOT humans[mh]) |  |
| 8 | #1 OR #2 OR #3 OR #4 | 1049161 |
| 9 | #5 AND #6 AND #7 AND #8 | 488 |
|  |  |  |
| **Embase datebase** | | |
| 1 | 'probiotics'/exp OR probiotic*:ab,ti OR Saccharomyce*:ab,ti OR Bifidobacteri*:ab,ti OR Lactobacill*:ab,ti OR Streptococc*:ab,ti OR Enterococc*:ab,ti OR Lactococc*:ab,ti OR 'Bacillus bacterium':ab,ti OR 'Escherichia coli':ab,ti OR Bacillus:ab,ti OR Clostridium:ab,ti OR 'Enterococcus faecalis':ab,ti OR Propionibacterium:ab,ti | 826228 |
| 2 | 'prebiotics'/exp OR prebiotic*:ab,ti OR inulin:ab,ti OR 'galactose oligosaccharide':ab,ti OR 'galactose oligomer':ab,ti OR oligogalactose:ab,ti OR fructan*:ab,ti OR  fructooligosaccharide*:ab,ti OR oligofructose:ab,ti OR galactooligosaccharide*:ab,ti OR  galacto-oligosaccharide*:ab,ti OR oligosaccharide*:ab,ti OR Idolax:ab,ti OR 'Raftilose P95':ab,ti | 74279 |
| 3 | 'synbiotics'/exp OR synbiotics:ab,ti OR synbioti*:ab,ti | 4357 |
| 4 | Postbiotic:ab,ti OR 'bacterial lysates':ab,ti OR Paraprobiotics:ab,ti OR 'Non-viable probiotics':ab,ti OR 'Heat-killed probiotics':ab,ti OR 'Tyndallized probiotics':ab,ti | 1366 |
| 5 | 'asthma'/exp OR asthma:ab,ti OR asthma*:ab,ti OR wheeze*:ab,ti OR wheezing*:ab,ti OR 'respiratory allergy*':ab,ti OR 'Bronchial Asthma':ab,ti | 385702 |
| 6 | 'Child'/exp OR Child:ab,ti OR child*:ab,ti OR 'Adolescent'/exp OR Adolescent:ab,ti OR adolescen*:ab,ti OR teens:ab,ti OR teen:ab,ti OR teenager*:ab,ti OR childhood:ab,ti OR youth*:ab,ti OR juvenile*:ab,ti OR minors:ab,ti | 5266902 |
| 7 | 'crossover procedure':de OR 'double-blind procedure':de OR 'randomized controlled trial':de OR 'single-blind procedure':de OR (random* OR factorial* OR crossover* OR cross NEXT/1 over* OR placebo* OR doubl* NEAR/1 blind* OR singl* NEAR/1 blind* OR assign* OR allocat* OR volunteer*):de,ab,ti |  |
| 8 | #1 OR #2 OR #3 OR # 4 | 882499 |
| 9 | #5 AND #6 AND #7AND #8 | 425 |
| **Cochrane Library** | | |
| 1 | MeSH descriptor: [probiotics] explode all trees OR (probiotic* or Saccharomyce* or Bifidobacteri* or Lactobacill* or Streptococc* or Enterococc*  or Lactococc* or Bacillus bacterium or Escherichia coli or Bacillus or Clostridium or Enterococcus faecalis or Propionibacterium):ti,ab,kw | 28486 |
| 2 | MeSH descriptor: [prebiotics] explode all trees OR (inulin OR galactose oligosaccharide OR galactose oligomer OR oligogalactose OR fructan* OR  fructooligosaccharide* OR fructo-oligosaccharide* OR oligofructose OR galactooligosaccharide* OR  galacto-oligosaccharide* OR oligosaccharide* OR Idolax OR Raftilose P95  ):ti,ab,kw | 3795 |
| 3 | MeSH descriptor: [synbiotics] explode all trees OR (synbiotics or synbioti*):ti,ab,kw | 1218 |
| 4 | (Postbiotics OR bacterial lysates OR Paraprobiotics OR Non-viable probiotics OR Heat-killed probiotics OR Tyndallized probiotics):ti,ab,kw | 434 |
| 5 | MeSH descriptor: [asthma] explode all trees  OR (asthma or asthma* or wheeze* or wheezing* or respiratory allergy* or Bronchial Asthma  ):ti,ab,kw | 41661 |
| 6 | MeSH descriptor: [Child] explode all trees OR (child* OR Adolescent OR adolescen* OR teens OR teen OR teenager* OR childhood OR youth* OR juvenile* OR minors  ):ti,ab,kw | 333333 |
| 7 | #1 OR #2 OR #3 OR # 4 | 31164 |
| 8 | #5 AND #6 AND #7 | 337 |
| **Web of Science** | | |
| 1 | TS=(probiotic*) OR TS=(lactobacillus) OR TS=(bifidobacterium) OR TS=(Saccharomyce*) OR TS=(Lactobacill*) OR TS=(Streptococc*) OR TS=(Lactococc*) OR TS=(Bacillus bacterium) OR TS=(Escherichia coli) OR TS=(Clostridium) OR TS=(enterococcus) OR TS=(Propionibacterium) OR TS=(prebiotic*) OR TS=(oligosaccharides) OR TS=(galacto oligosaccharide) OR TS=(galactooligosaccharide) OR TS=(galactose oligomer) OR TS=(oligogalactose) OR TS=(galactooligosaccharides) OR TS=(fructooligosaccharides) OR TS=(fructan* ) OR TS=(synbiotic*) OR TS=(synbiotics) OR TS=(Postbiotics) OR TS=(bacterial lysates) OR TS=(Paraprobiotics) OR TS=(Non-viable probiotics) OR TS=(Heat-killed probiotics) OR TS=(Tyndallized probiotics) | 6889 |
| 2 | TS=(asthma) OR TS=(asthma*) OR TS=(wheeze*) OR TS=(wheezing*) OR TS=(respiratory allergy*) OR TS=(Bronchial Asthma) | 18214 |
| 3 | TS=(Child) OR TS=(children) OR TS=(childhood) OR TS=(Adolescen*) OR TS=(Adolescent) OR TS=(Teens) OR TS=(Teen) OR TS=(Teenagers) OR TS=(Teenager) OR TS=(Youth) OR TS=(Youths) OR TS=(juvenile) OR TS=(juveniles) OR TS=(minors) | 1057760 |
| 4 | TS=(randomized controlled trial) OR TS=(Controlled clinical trial) OR TS=(Random*) OR TS=(controlled) OR TS=(Trial) OR TS=(blind) OR TS=(Parallel group) OR TS=(trials) | 1176066 |
| 5 | #1 AND #2 AND #3 AND #4 | 11 |
